# 2018–2019 field seasons of the Maize Genomes to Fields (G2F) G x E project

**DOI:** 10.1186/s12863-023-01129-2

**Published:** 2023-05-25

**Authors:** Dayane Cristina Lima, Alejandro Castro Aviles, Ryan Timothy Alpers, Bridget A. McFarland, Shawn Kaeppler, David Ertl, Maria Cinta Romay, Joseph L. Gage, James Holland, Timothy Beissinger, Martin Bohn, Edward Buckler, Jode Edwards, Sherry Flint-Garcia, Candice N. Hirsch, Elizabeth Hood, David C. Hooker, Joseph E. Knoll, Judith M. Kolkman, Sanzhen Liu, John McKay, Richard Minyo, Danilo E. Moreta, Seth C. Murray, Rebecca Nelson, James C. Schnable, Rajandeep S. Sekhon, Maninder P. Singh, Peter Thomison, Addie Thompson, Mitchell Tuinstra, Jason Wallace, Jacob D. Washburn, Teclemariam Weldekidan, Randall J. Wisser, Wenwei Xu, Natalia de Leon

**Affiliations:** 1 Department of Agronomy, University of WI – Madison, Madison, WI 53706 USA; 2 BASF Vegetable Seeds, Brooks, OR 97305 USA; 3 Panama-USA Commission for the Eradication and Prevention of Screwworm (COPEG), USDA-APHIS-IS, Pacora, Panama; 4 Iowa Corn Promotion Board, Johnston, IA 50131 USA; 5 Institute for Genomic Diversity, Cornell University, Ithaca, NY 14853 USA; 6 Department of Crop and Soil Sciences, North Carolina State University, Raleigh, NC 27695 USA; 7 USDA-ARS Plant Science Research Unit, Raleigh, NC 27606 USA; 8 Department of Crop Science, University of Göttingen Center for Integrated Breeding Research, Carl-Sprengel-Weg 1, 37075 Göttingen, Germany; 9 University of Illinois at Urbana-Champaign, Urbana, IL 61801 USA; 10 USDA-ARS and Cornell University, Ithaca, NY 14853 USA; 11 USDA ARS CICGRU, 716 Farmhouse Ln, Ames, IA 50011-1051 USA; 12 USDA-ARS, Columbia, MO 65211 USA; 13 Department of Agronomy and Plant Genetics, University of Minnesota, St Paul, MN 55108 USA; 14 College of Agriculture, Arkansas Biosciences Institute, Arkansas State University, Jonesboro, AR 72404 USA; 15 Department of Plant Agriculture, University of Guelph, Ridgetown Campus, Ridgetown, ON Canada; 16 USDA-ARS Crop Genetics and Breeding Research Unit, Tifton, GA 31793 USA; 17 School of Integrative Plant Science, Cornell University, Ithaca, NY 14850 USA; 18 Department of Plant Pathology, Kansas State University, Manhattan, KS 66503 USA; 19 Department of Agricultural Biology, Colorado State University, Fort Collins, CO 80523 USA; 20 Department of Horticulture and Crop Science, Ohio State University College of Food, Agricultural, and Environmental Sciences, Wooster, OH 44691 USA; 21 Department of Soil and Crop Sciences, Texas A&M University, College Station, TX 77843 USA; 22 Cornell University, Ithaca, NY 14853 USA; 23 Department of Agronomy and Horticulture, University of Nebraska-Lincoln, Lincoln, NE 68588 USA; 24 Department of Genetics and Biochemistry, Clemson University, Clemson, SC 29634 USA; 25 Department of Plant, Soil and Microbial Sciences, Michigan State University, East Lansing, MI 48824 USA; 26 Ohio State University, Columbus, OH 43210 USA; 27 Department of Agronomy, Purdue University, West Lafayette, IN 49707 USA; 28 Department of Crop & Soil Sciences, University of Georgia, Athens, GA 30602 USA; 29 Department of Plant and Soil Sciences, University of Delaware, Newark, DE 19716 USA; 30 Laboratoire d’Ecophysiologie Des Plantes Sous Stress Environmentaux, INRAE, 34060 Montpellier, France; 31 Texas A&M University, College Station, TX 77843 USA

**Keywords:** Maize, Genotype by environment, Phenotype, Variable environments, Grain yield

## Abstract

**Objectives:**

This report provides information about the public release of the 2018–2019 Maize G X E project of the Genomes to Fields (G2F) Initiative datasets. G2F is an umbrella initiative that evaluates maize hybrids and inbred lines across multiple environments and makes available phenotypic, genotypic, environmental, and metadata information. The initiative understands the necessity to characterize and deploy public sources of genetic diversity to face the challenges for more sustainable agriculture in the context of variable environmental conditions.

**Data description:**

Datasets include phenotypic, climatic, and soil measurements, metadata information, and inbred genotypic information for each combination of location and year. Collaborators in the G2F initiative collected data for each location and year; members of the group responsible for coordination and data processing combined all the collected information and removed obvious erroneous data. The collaborators received the data before the DOI release to verify and declare that the data generated in their own locations was accurate. ReadMe and description files are available for each dataset. Previous years of evaluation are already publicly available, with common hybrids present to connect across all locations and years evaluated since this project’s inception.

## Objective

Maize (*Zea mays* subsp. *mays* L.) plays an important role in the global economy. As a crop, it displays a variety of uses such as food, feed, and fuel. At the same time, and due to its versatility and relevance, maize has been widely studied. The Genomes to Fields (G2F) is a collaborative initiative involving scientists from the public sector that support growers, consumers, and society. G2F researchers generate phenotypic, genotypic, environmental, and metadata datasets to facilitate the understanding of the potential and challenges of maize production in different environments.

Individual genotype performances differ across environments, and the magnitude of this difference dictates the importance of the Genotype by Environment (G × E) interaction. Understanding and harnessing G × E interactions improves the efficiency in the use and allocation of resources, and it facilitates the identification of genotypes with higher stability across a range of locations, the identification of locations where the effect of G x E is minimized, and the identification of mechanisms affecting the differential response of phenotypes to variable environments. Furthermore, advances in our understanding of the fundamental components contributing to the differential response of plants to environmental cues will also improve genomic and phenotypic predictabilities for traits of interest. Therefore, this data release provides a unique resource of combined agronomic, phenological, and morphological information to dissect G × E interaction.

In the 2018 and 2019 experiments, 1153 publicly available hybrids were evaluated through a network of collaborators in 32 different locations. The main group of hybrids was produced by the cross of doubled-haploid (DH) inbred lines from a collection of three biparental populations that share one parent in common (PHW65) and PHN11, Mo44, and MoG as the alternative parent, to two ex-PVP inbred testers, LH195 in Midwest to Southern locations, and PHT69 in Northern locations.

## Data description

The 2018 and 2019 datasets are publicly available via CyVerse/iPlant and structured as described in Table 1. Briefly, the datasets included here are:

- **Phenotypic dataset:** Phenotypic measurements that follow a standard set of instructions, available in the G2F webpage [1]. Standard traits include days to anthesis, days to silking, ear height, plant height, stand count, stalk lodging, root lodging, grain moisture, test weight, plot weight, and estimated grain yield. Raw data and quality-controlled data are reported. Out of range observations were set to missing following the rules described in the readMe and data description files.
- **Genotypic dataset:** Inbred parents of the tested hybrids were genotyped using the Practical Haplotype Graph (PHG) [2, 3]. The data is minimally filtered, allowing the public to perform their own quality control steps prior to using it. The raw sequencing reads are available under BioProject ID PRJNA530187 [4]. The code used to create the genotypic data is also available at https://bitbucket.org/bucklerlab/g2f_2018_phg_genotyping/src/master/.
- **Environmental dataset:** WatchDog 2700 weather stations (Spectrum Technologies) were placed at each field site. Data was collected at 30-min intervals from planting through harvest at each location. The geographic locations of the experiments are not identical across years due to crop rotation management practices; thus, the locations of the weather stations vary across years. Each station measured wind speed, direction, and gust; air temperature, dewpoint, relative humidity; soil temperature and moisture; rainfall and solar radiation. Additional measurements taken at selected sites included soil electrical conductivity, ultra-violet light, carbon dioxide, and photosynthetically active radiation. Instructions for weather station maintenance activities including pre-season tasks, field setup, maintenance throughout the growing season, and removal are available in the G2F webpage [5].
- **Soil dataset:** Each field location collected soil samples that represent the experiment field. Collaborators follow instructions available on the G2F webpage for sample collection [5].
- **Supplemental dataset:** Supplemental information consists of metadata (any field-level data collected at planting, in season, and/or at harvest), agronomic information (list of pesticides, nutrients, and irrigation applied), and cooperator list (collaborators responsible for the field locations in 2018 and 2019).

**Table 1:** Overview of data files and dataset for 2018 and 2019 planting seasons

Label	Name of data file/data set	File types (Extension)	Data repository and identifier (DOI or accession number)
Data set 1	Evaluation of genetic diversity across the inbreds used by G2F project (WGS skim sequencing)	fastq files (.fq.gz)	NCBI *BioProject* (https://identifiers.org/ncbi/bioproject:PRJNA530187) [4]
Data file 1	README.txt	Text file (.txt)	CyVerse (https://doi.org/10.25739/anqq-sg86) [6]
Data file 2	README.txt	Text file (.txt)	CyVerse (https://doi.org/10.25739/anqq-sg86) [6]
Data file 3	_g2f_2018_hybrid_data_description.pdf	Portable Document Format (.pdf)	CyVerse (https://doi.org/10.25739/anqq-sg86) [6]
Data file 4	g2f_2018_hybrid_data_clean.csv	comma-separated values file (.csv)	CyVerse (https://doi.org/10.25739/anqq-sg86) [6]
Data file 5	g2f_2018_hybrid_data_raw.csv	comma-separated values file (.csv)	CyVerse (https://doi.org/10.25739/anqq-sg86) [6]
Data file 6	raw_cleaning_readme.txt	Text file (.txt)	CyVerse (https://doi.org/10.25739/anqq-sg86) [6]
Data file 7	README_weather.txt	Text file (.txt)	CyVerse (https://doi.org/10.25739/anqq-sg86) [6]
Data file 8	_g2f_2018_weather_data_description.pdf	Portable Document Format (.pdf)	CyVerse (https://doi.org/10.25739/anqq-sg86) [6]
Data file 9	g2f_2018_weather_clean.csv	comma-separated values file (.csv)	CyVerse (https://doi.org/10.25739/anqq-sg86) [6]
Data file 10	g2f_2018_weather_raw.csv	comma-separated values file (.csv)	CyVerse (https://doi.org/10.25739/anqq-sg86) [6]
Data file 11	weather_cleaning_readme.txt	Text file (.txt)	CyVerse (https://doi.org/10.25739/anqq-sg86) [6]
Data file 12	Indigo_2018_Soil_Data.pdf	Portable Document Format (.pdf)	CyVerse (https://doi.org/10.25739/anqq-sg86) [6]
Data file 13	_g2f_2018_soil_description.pdf	Portable Document Format (.pdf)	CyVerse (https://doi.org/10.25739/anqq-sg86) [6]
Data file 14	g2f_2018_soil_data.csv	comma-separated values file (.csv)	CyVerse (https://doi.org/10.25739/anqq-sg86) [6]
Data file 15	G2F_PHG_minreads1_Mo44_PHW65_MoG_assemblies_14112019_filtered_plusParents.vcf	variant call format file (.vcf)	CyVerse (https://doi.org/10.25739/anqq-sg86) [6]
Data file 16	G2F_PHG_minreads1_Mo44_PHW65_MoG_assemblies_14112019_filtered_plusParents_description.pdf	Portable Document Format (.pdf)	CyVerse (https://doi.org/10.25739/anqq-sg86) [6]
Data file 17	G2F_PHG_minreads1_Mo44_PHW65_MoG_assemblies_14112019_filtered_plusParents_sampleDecoder.txt	Text file (.txt)	CyVerse (https://doi.org/10.25739/anqq-sg86) [6]
Data file 18	README_G2F_2020-03–13.txt	Text file (.txt)	CyVerse (https://doi.org/10.25739/anqq-sg86) [6]
Data file 19	README_Genotypic.txt	Text file (.txt)	CyVerse (https://doi.org/10.25739/anqq-sg86) [6]
Data file 20	g2f_2018_agronomic information.csv	comma-separated values file (.csv)	CyVerse (https://doi.org/10.25739/anqq-sg86) [6]
Data file 21	g2f_2018_cooperators_list.csv	comma-separated values file (.csv)	CyVerse (https://doi.org/10.25739/anqq-sg86) [6]
Data file 22	g2f_2018_field_metadata.csv	comma-separated values file (.csv)	CyVerse (https://doi.org/10.25739/anqq-sg86) [6]
Data file 23	g2f_2018_supplemental_information.txt	Text file (.txt)	CyVerse (https://doi.org/10.25739/anqq-sg86) [6]
Data file 24	g2f_planting_season_2019_readMe.txt	Text file (.txt)	CyVerse (https://doi.org/10.25739/t651-yy97) [7]
Data file 25	g2f_2019_phenotypic_clean_data.csv	comma-separated values file (.csv)	CyVerse (https://doi.org/10.25739/t651-yy97) [7]
Data file 26	g2f_2019_phenotypic_data_description.pdf	Portable Document Format (.pdf)	CyVerse (https://doi.org/10.25739/t651-yy97) [7]
Data file 27	g2f_2019_phenotypic_data_read_me.txt	Text file (.txt)	CyVerse (https://doi.org/10.25739/t651-yy97) [7]
Data file 28	g2f_2019_phenotypic_raw_data.csv	comma-separated values file (.csv)	CyVerse (https://doi.org/10.25739/t651-yy97) [7]
Data file 29	2019_weather_cleaned.csv	comma-separated values file (.csv)	CyVerse (https://doi.org/10.25739/t651-yy97) [7]
Data file 30	2019_weather_raw.csv	comma-separated values file (.csv)	CyVerse (https://doi.org/10.25739/t651-yy97) [7]
Data file 31	g2f_2019_weather_data_description.pdf	Portable Document Format (.pdf)	CyVerse (https://doi.org/10.25739/t651-yy97) [7]
Data file 32	g2f_2019_weather_readMe.txt	Text file (.txt)	CyVerse (https://doi.org/10.25739/t651-yy97) [7]
Data file 33	g2f_2019_soil_data.csv	comma-separated values file (.csv)	CyVerse (https://doi.org/10.25739/t651-yy97) [7]
Data file 34	g2f_2019_soil_data_description.pdf	Portable Document Format (.pdf)	CyVerse (https://doi.org/10.25739/t651-yy97) [7]
Data file 35	g2f_2019_agronomic_information.csv	comma-separated values file (.csv)	CyVerse (https://doi.org/10.25739/t651-yy97) [7]
Data file 36	g2f_2019_cooperators_list.csv	comma-separated values file (.csv)	CyVerse (https://doi.org/10.25739/t651-yy97) [7]
Data file 37	g2f_2019_field_metadata.csv	comma-separated values file (.csv)	CyVerse (https://doi.org/10.25739/t651-yy97) [7]
Data file 38	g2f_2019_supplemental_information.pdf	Portable Document Format (.pdf)	CyVerse (https://doi.org/10.25739/t651-yy97) [7]

### Limitations

These datasets contain missing data. Missing data includes data not reported by collaborators or erroneous data as determined by data description files. In 2019, some locations had pedigree information set to missing due to packaging problems and only plot number was reported in the phenotypic dataset to reduce misinterpretation.

## Data Availability

The data described in this Data note can be freely and openly accessed on CyVerse at https://doi.org/10.25739/anqq-sg86 (2018 Field Season) and https://doi.org/10.25739/t651-yy97 (2019 Field Season). Please see Table 1 and references list for details and links to the data.

## Abbreviations

G2F: Genomes to Fields
DOI: Digital Object Identifier
DH: Doubled-haploid
G x E: Genotype by environment
